# The will to live, illness representations, hope, and heart failure self-care

**DOI:** 10.3389/fcvm.2026.1899596

**Published:** 2026-10-12

**Authors:** Carolyn Kleman, Kayla Alderson, Chris Evonko, Caroline Jenkins, Nia Lewis, Stephanie Turrise

**Affiliations:** School of Nursing, University of North Carolina Wilmington, Wilmington, NC, United States

**Keywords:** heart failure, hope, illness representations, self-care, will to live

## Abstract

**Background:**

Heart failure (HF) remains one of the most prevalent cardiovascular diseases worldwide. Effective management requires both medical treatment and patient engagement in self-care activities. Because self-care can be challenging to sustain, understanding the factors that are associated with self-care behaviors is essential. Illness threat, coping resources, and self-care efficacy may affect heart failure self-care (HFSC). The will to live (WTL) and hope, as coping-related resources, may motivate individuals with HF to engage in self-care and strengthen their confidence in managing their condition.

**Objective:**

To examine the associations among illness threat, will to live, hope, and heart failure self-care.

**Methods:**

Moderation analyses and multiple regression were conducted using survey data from 204 adults with heart failure.

**Results:**

Illness threat did not significantly influence HFSC and was not moderated by WTL or hope. In contrast, both WTL and hope were positively associated with HFSC and self-care efficacy.

**Conclusion:**

Across all HFSC domains—maintenance, monitoring, management, and self-care efficacy—WTL and hope emerged as consistent and significant correlates. Illness threat showed weak or non-significant associations with HFSC and did not interact with WTL or hope in predicting self-care behaviors. These findings suggest that coping and dispositional resources may play a more central role in HFSC engagement than illness perceptions alone. Interventions that focus primarily on emphasizing illness threat may be less effective than those that assess and strengthen patients’ hope, will to live, and sense of agency in managing their condition.

Heart failure (HF) is a life-limiting chronic condition that affects over 6.7 million Americans, straining patients, families, and health systems (1). Self-care is essential for effective HF management and requires individuals to consistently perform daily HF self-care (HFSC) behaviors such as taking medications, weighing, exercising, and monitoring symptoms. Current guidelines have acknowledged the importance of self-care in reducing unplanned hospital readmissions, decreasing mortality rates, and improving health outcomes among people with HF (2–4). However, despite the known benefits and the need to practice HFSC behaviors, their adoption remains suboptimal. In a systematic review with meta-analysis of 39 studies conducted in 12 countries, the average scores for self-care maintenance, self-care management, and self-care efficacy were all in the 50's (5). These scores are well below the 70-cut point that indicates adequate, not optimal, self-care (6). This is not a unique problem to the United States. Worldwide, others have also reported poor uptake of HFSC behaviors (7–10) despite advances in therapies and our understanding of what is needed to optimize HF outcomes. Contributing factors to insufficient self-care behaviors include low self-efficacy, lack of social support, lower education and health literacy levels, and “poor” illness perceptions (11, 12). Furthermore, in a recent qualitative study investigating readiness to engage in HFSC, Turrise and colleagues (13) identified the will to live as a theme that emerged across all participants (*n* = 21). The will to live or lack of it was a driving force in participants performing, or not, their healthcare professionals' HFSC recommendations.

## Theoretical framework

### Common sense model of illness self-regulation (CSM)

The Common-Sense Model of Illness Self-regulation (CSM), recently updated (14), reflects current chronic illness management, moving away from the inpatient setting toward self-managing complex, and in many cases, multiple chronic conditions at home. Individuals and families bear an increasing burden of responsibility for their own treatment. Successful treatment outcomes are, therefore, increasingly dependent on one's capacity to self-regulate. The CSM theory states that individuals develop illness representations, cognitive and emotional, in response to an illness or “health threat”. To manage the illness, individuals rely on their cognitive representations, how they perceive their illness, and their emotional representations, or how they feel about their illness, to guide planning and actions (14). Additionally, the theory posits that individuals use coping strategies (action or avoidant) rooted in their beliefs about behaviors and the cognitive and emotional representations they formed about their illness. Once actions are implemented, they evaluate the effectiveness of their plans and coping strategies based on the outcome and revise their understanding and plans, resulting in successive cycles of self-regulation (14, 15). See Figure 1. Snyder (16) defines hope as…“a positive motivational state that is based on an interactively derived sense of successful goal-directed energy and planning to meet goals.” Hope is more than wishful thinking; it is action-oriented. The will to live, is the profound, often unconscious, psychological and instinctual drive for self-preservation and survival, particularly when faced with mortality, disease, or intense adversity. These dispositional attributes, hope and will to live, may be fundamental coping mechanisms, emotional drivers that guide the behaviors people with HF use to manage their illness or health threat.

**Figure 1:**
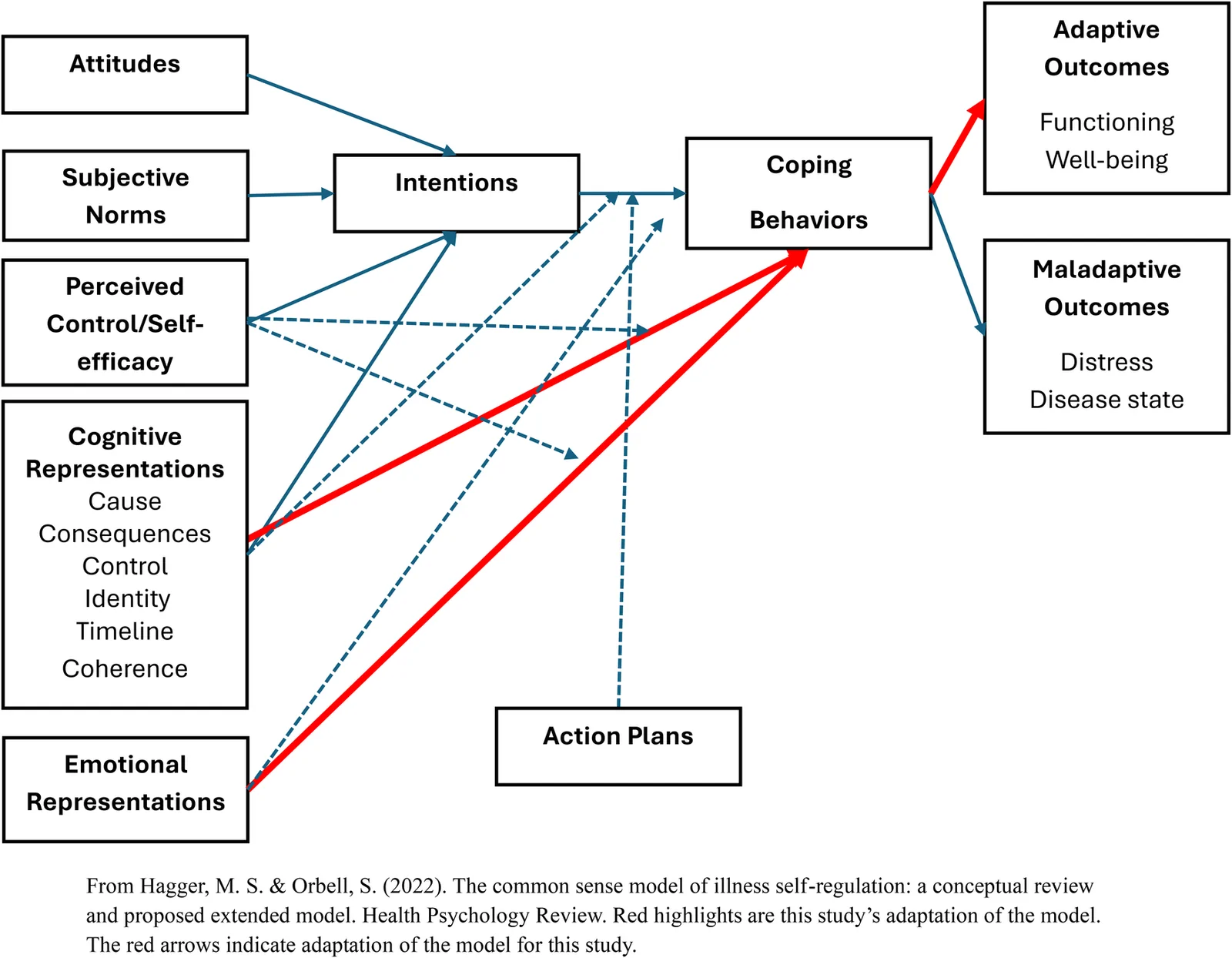
Common sense model.

Within the CSM framework, hope is a coping mechanism that can influence HFSC behavior (17), According to Snyder's Hope Theory, hope consists of two components: agency and pathways (16). Agency is one's perceived ability and determination to pursue a goal while pathways represent one's capacity to identify strategies and plan to achieve goals (16).

The will to live (WTL) is also a psychosocial coping construct that is a crucial indicator of well-being in chronic illnesses (18, 19). Research suggests that the WTL contributes to a patient's readiness, commitment, and capacity regarding their engagement in HFSC (13). Hope and WTL have both been studied in chronic illness and cardiovascular disease specifically (17, 19–22). However, the relationships among hope, will to live, illness representations, and HFSC remain unexplored. See Figure 1.

### Adaptive outcomes based on the situation specific theory of heart failure self-care

The Situation Specific Theory of Heart Failure Self-Care was recently updated (2022) and suggests that interactions among the person, environment, and problems encountered create a context in which self-care decisions are made. Self-care is essential for effective HF management and requires consistently performed HF self-care (HFSC) behaviors that include self-care (SC) maintenance, SC monitoring, and SC management (23). Self-care maintenance refers to routine behaviors such as medication adherence, sodium restriction, and daily exercise (23). SC monitoring includes continuous observation of changes in signs and symptoms of HF such as tracking weight gain and blood pressure (23). SC management is the actions taken in response to symptom changes (23). In all components of HFSC, SC self-efficacy is crucial because it refers to one's confidence in the ability to perform the actions needed to manage their HF (24, 25). In all components of HFSC, self-efficacy is crucial because it reflects one's confidence in their ability to perform the actions needed to manage their HF (24, 25).

### Study design

This study used a cross-sectional design to identify associations between variables. The cross-sectional design allowed researchers to capture variable data at one point in time so that relationships among variables could be measured and described. A moderation model was designed to measure the strength of the relationship between illness representation with hope and will to live as moderators of the relationship between illness representation and HFSC. See Figure 2.

**Figure 2:**
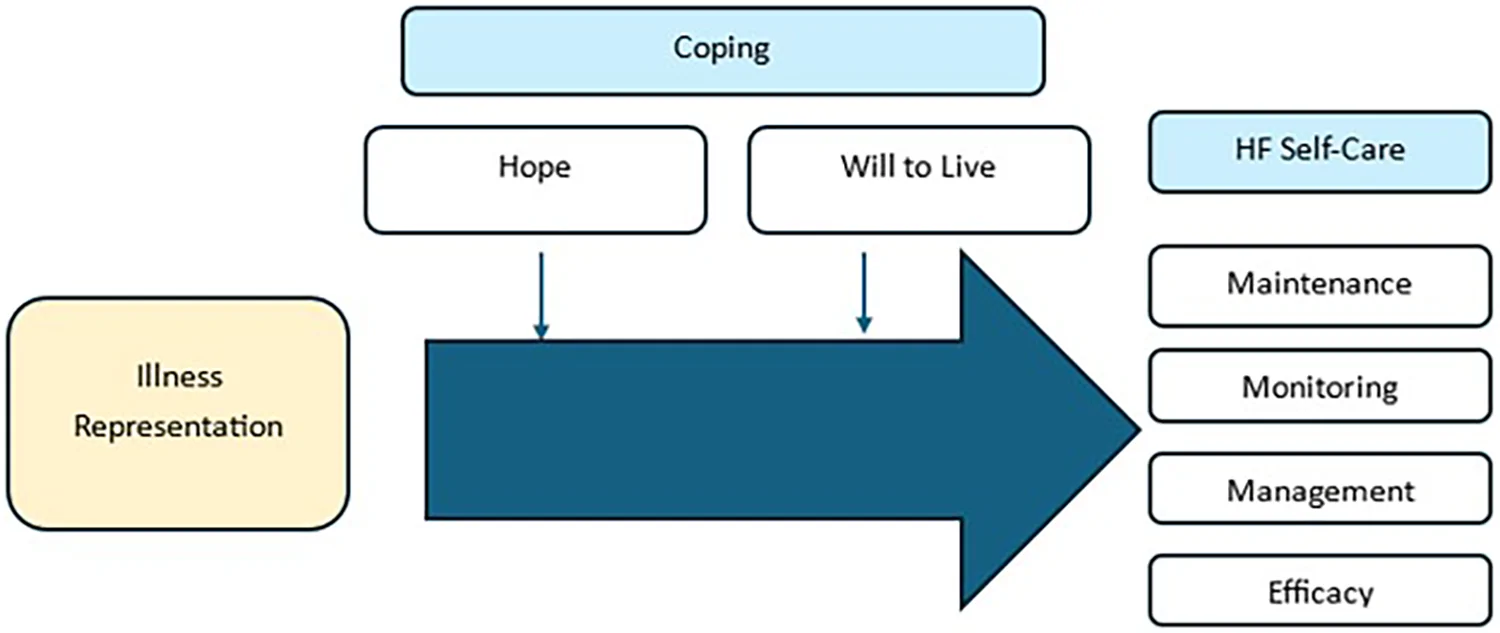
Initial moderation model 1.

### Participants

Convenience sampling was used in this study. Inclusion criteria included participants must be age 18 and older, able to read and understand English, live in the United States, have a diagnosis of and were being treated for heart failure. Exclusion criteria consisted of those not consenting to participate and/or did not pass the attention checks. Human subjects' protection review was granted by the University of North Carolina Wilmington Institutional Review Board # H25-0217.

Prolific (https://www.prolific.com), a platform that helps researchers recruit participants for their online research was used to recruit participants throughout the United States. Participants using the Prolific website to access research questionnaires complete demographic and history questions by Prolific. Thus, only those who had indicated that they have a cardiovascular condition including a diagnosis of HF and were being treated for it were able to view and access the study. Participants remain anonymous by using their Prolific ID and no identifying information is collected. Additionally, potential participants are able to review a brief overview of the research purpose, approximate length for completion of the study, and the incentive for completion. If interested, they clicked a link that takes them to a Qualtrics survey. Prior to screening and participating in the survey, participants are provided consent and must agree to participate. Once they consented, to verify inclusion/exclusion criteria, participants answered screening questions. Participants were directed to a Qualtrics survey from Prolific. Recruitment and data collection occurred during the month of February 2025. Prolific identified the pool of potential participants based on the inclusion criteria and sent an invitation to participate. Out of 724 potential participants, 234 were recruited and agreed to participate with 204 participants included in the study. Oversampling was done to account for potential attrition and exclusion criteria having not been met. See Figure 3. Sample size estimate of 147 was based on a moderate effect size (based on theory and evidence), *α* = 0.01, *β* = 0.80, using Cohen's guidelines (26).

**Figure 3:**
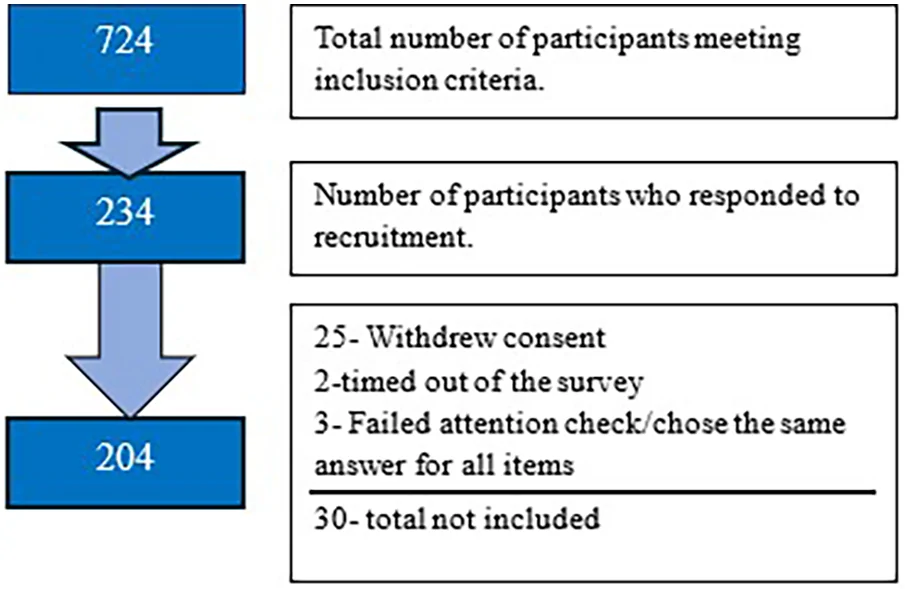
Participant flow diagram.

### Data sources/measurement

#### Illness representations (BIPQ)

The Brief Illness Perception Questionnaire (27, 28) is used to measure illness representations. There are eight Likert-type questions, each representing a specific dimension of illness representation. Each item is rated 1 (no effect at all) to 10 (severely affects my life). A total score indicates how threatening an individual perceives their illness and a possible total score ranging from 0 to 80 with a score <42 indicating low experienced threat, 42–49 moderate experienced threat, and ≥50 high experienced threat (29). The last item is a qualitative question asking individuals to “list in rank-order the three most important factors thou believe caused your illness” and was not asked in the current study. Initial reliability (Cronbach's alpha) of each item was 0.63–0.72 and good test-retest reliability has been demonstrated (27).

#### Will to live (WTLS)

The WTL Scale (30) has five Likert-type questions with a scale of 0 (I have no will to live) to 5 (much stronger will to live). The total possible score is 0–25 with higher scores indicating a stronger will to live. The WTL tool is valid and reliable (30). In the initial study with three data collection times, Cronbach's alpha was 0.83, 0.86, 0.90, respectively. Correlations with other well-established indicators were established: life satisfaction, happiness, and self-rated health. The correlation was negative for depression and loneliness. The Cronbach's alpha reliability score was 0.60.

#### Hope (AHS)

The Trait Hope Scale (also referred to as the Adult Trait Hope Scale) is a valid, reliable two-factor scale that represents agency (motivation) and pathways (planning or strategizing) toward goal attainment (16). A total of 12 items with Likert-type scaling of 1 (definitely false) to 8 (definitely true) with a range of 8–64. Four of the items are considered filler items. The Cronbach's alpha reliability score was 0.67 in this study.

#### Heart failure self-care (SCHFI)

The Self-Care of Heart Failure Index (SCHFIv8.2) (31) is a widely used, valid, and reliable scale measuring three domains: self-care (SC) maintenance, SC monitoring, and SC management. The subscales have 8, 7, and 8 Likert-type items each, respectively. A range of scores for each is 8–40, 7–35, and 8–40, respectively. The Cronbach's alpha reliability scores in this study were SC maintenance 0.75, SC maintenance 0.75, SC monitoring 0.84.

#### The self-care self-efficacy (SCSE) scale

Self-care self-efficacy is an additional domain that has been empirically recognized as a key predictor to successful self-care. The scale, originally part of the SCHFI, is now a separate measure and is highly recommended by the authors of the SCHFI when measuring HFSC (32). The scale is 10 Likert-type questions, with a range of 1 (not confident) to 5 (extremely confident) in carrying out self-care behaviors. The scoring range is 10–50. Cronbach's alpha reliability score was 0.87.

## Results

### Sociodemographic characteristics

A total of 204 participants were included, with 114 males and 90 females. The mean age of the sample was 64.76. Over 70% of participants were college-educated and another 11% completed some college-level work but did not graduate. The majority of participants were Caucasian (73.5%), employed (69.6%), and made over $75,000 a year (55%). See Table 1 for further details.

**Table 1:** Demographic characteristics.

Demographic characteristic	Total *n* = 204	Percent
Age range in years	47–92	—
Mean age in years	64.76	—
Gender		
Male	114	56%
Female	90	44%
Race		
Black or African American	42	20.5%
American Indian	1	0.5%
Caucasian	150	73.5%
Asian	4	2%
Native Hawaiian or Other Pacific Islander	2	1%
Two or more races	3	1.5%
Prefer not to answer	2	1%
Ethnicity		
Hispanic or Latino	23	11%
Not Hispanic or Latino	175	86%
Prefer not to answer	6	3%
Education		
Graduate professional	83	41%
Bachelor's degree	64	31%
Some college but did not graduate	23	11%
Associate's degree	16	8%
High school graduate or GED	14	7%
Some high school but did not graduate high school	2	1%
Vocational or technical school completed	1	0.5%
Prefer not to answer/missing data	1	0.5%
Months with heart failure		
Mean	5.8 years	
Range	6 months–30 years	
Employment status		
Employed	142	69.6%
Unemployed	9	4.4%
Retired	30	14.7%
Disabled, unable to work	17	8.3%
Homemaker	1	0.5%
Student	4	2%
Prefer not to answer	1	0.5%
Annual income		
$10,000–$25,000	24	18%
$25,001–$50,000	34	16%
$50,001–$75,000	31	15%
$75,001–$100,000	38	19%
Over $100,000	74	36%
Prefer not to answer income question/ missing data	3	1.5%
Health Insurance		
Yes	196	96%
No	5	2.5%
Prefer not to answer	3	1.5%
Number of Comorbidities		
Range	1–23	
Mean	3.63	
Marital Status		
Divorced	14	7.5%
Single	29	14%
Married/Domestic partner	120	60%
Living with a partner in a committed relationship	19	9%
Serious/committed relationship but not living together	5	2.5%
Widowed	13	5%
Separated	2	1%
Prefer not to answer	2	1%

### Correlation analysis among variables

As seen in Table 2, there are many positive correlations between variables with the highest correlations (>0.50) between SC management and SC maintenance (0.55), SC management and SC monitoring (0.58), SC maintenance and SC monitoring (0.59), SC efficacy and SC management (0.55), and SC efficacy and SC maintenance (0.61). We would expect to see higher correlations between SC management, maintenance, and monitoring and SC efficacy as they are typically highly correlated (25). Of note, WTL and SC Management (0.41) and Hope and SC efficacy (0.43) were moderately correlated. In Table 3, the questionnaire scores that the correlations were based on are detailed. Also, of note, illness representation (illness threat) was weakly correlated with all other variables.

**Table 2:** Pearson correlation analysis among variables.

Variable	1	2	3	4	5	6	7
1. Illness Threat BIPQ Total	—						
2. Will to Live WTLS Total	0.19**	—					
3. Hope AHS Total	0.07	0.29***	—				
4. SC Management	−0.02	0.41***	0.38***	—			
5. SC Maintenance	−0.15*	0.31***	0.33***	0.55***	—		
6. SC Monitoring	0.12	0.21***	0.36***	0.58***	0.59***	—	
7. Self-Care Efficacy SCES Total	−0.16*	0.38***	0.43***	0.55***	0.61***	0.16*	—

Em dash (—) indicates a variable correlated with itself.

*p < 0.05, **p < 0.01, ***p < 0.001.

**Table 3:** Questionnaire scores.

Questionnaire scores	Mean	SD	Min	Max	Range
BIPQ Total (Illness Threat)	42.11	10.07	11.57	68	56.43
Hope Total	49.45	8.83	22.46	64	41.54
SC Efficacy	78.00	15.61	31.11	100	68.89
SC Maintenance	73.18	14.00	31.25	100	68.75
SC Management	71.31	18.58	14.77	100	85.23
SC Monitoring	73.55	15.54	25.47	100	74.53
Will to Live Total	3.74	0.82	1.22	5	3.78

### Moderation analysis

Moderation analysis was conducted based on Model 1. See Figure 3. Assumptions were checked and no violations were found.

## Findings

### Heart failure self-care

#### Maintenance

In evaluating the association between illness threat and self-care maintenance, the effect of illness threat was trending but did not reach statistical significance (*β* = −0.17, SE = 0.10, *p* = 0.055). A significant effect of hope was observed on self-care maintenance (*β* = 0.51, SE = 0.10, *p* < 0.001). Greater levels of hope were associated with greater self-care maintenance. The overall model was significant and explained roughly 12% of the variance [*F*(3, 201) = 10.23, *p* < 0.001]. In evaluating the effects of will to live, a significant association with SC maintenance was observed (*β* = 5.18, SE = 1.18, *p* < 0.001). Greater levels of WTL were associated with greater levels of SC maintenance. The overall model was statistically significant [*F*(3, 201) = 8.13, *p* < 0.001], explaining roughly 10% of the variance. No significant interaction of hope or WTL was observed on the relationship between illness threat and SC maintenance (*p* > 0.01).

#### Management

In evaluating the association between illness threat and self-care management, there was no significant effect of illness threat (*β* = 0.01, SE = 0.12, *p* = 0.962). A significant effect of hope was observed on SC management (*β* = 0.81, SE = 0.14, *p* < 0.001). Greater levels of hope were associated with greater levels of SC management. The overall model was significant and explained roughly 14% of the variance [*F*(3, 201) = 11.72, *p* < 0.001]. In evaluating the effects of will to live, a significant association with SC management was observed (*β* = 9.68, SE = 1.50, *p* < 0.001). Greater levels of will to live were associated with greater levels of SC management. The overall model was statistically significant, explaining roughly 16% of the variance [*F*(3, 201) = 14.26, *p* < 0.001]. No significant interaction of hope or WTL were observed on the relationship between illness threat and SC monitoring (*p* > 0.01).

#### Monitoring

In evaluating the association between illness threat and self-care monitoring, the effect of illness threat was trending but did not reach significance (*β* = 0.22, SE = 0.10, *p* = 0.025). A significant effect of hope was observed on SC monitoring (*β* = 0.65, SE = 0.11, *p* < 0.001). Greater levels of hope were associated with greater levels of SC monitoring. The overall model was significant and explained roughly 14% of the variance [*F*(3, 201) = 12.22, *p* < 0.001]. In evaluating the effects of will to live, a significant association with SC monitoring was observed (*β* = 4.77, SE = 1.33, *p* < 0.001). Greater levels of will to live were associated with greater levels of SC monitoring. The overall model was statistically significant, explaining roughly 6% of the variance [*F*(3, 201) = 5.37, *p* < 0.010]. No significant interaction of hope or WTL were observed on the relationship between illness threat and SC monitoring (*p* > 0.01).

#### Efficacy

In evaluating the association between illness threat and self-care efficacy, the effect of illness threat was trending but did not reach significance (*β* = −0.21, SE = 0.10, *p* = 0.032). A significant effect of hope was observed on SC efficacy (*β* = 0.74, SE = 0.11, *p* < 0.001). Greater levels of hope were associated with greater levels of SC monitoring. The overall model was significant and explained roughly 19% of the variance [*F*(3, 201) = 17.29, *p* < 0.001]. In evaluating the effects of will to live, a significant association with SC efficacy was observed (*β* = 6.92, SE = 1.28, *p* < 0.001). Greater levels of will to live were associated with greater levels of SC efficacy. The overall model was statistically significant, explaining roughly 14% of the variance [*F*(3, 201) = 12.30, *p* < 0.001]. No significant interaction of hope or WTL was observed on the relationship between illness threat and SC efficacy (*p* > 0.01).

Based on these statistical findings, illness threat (illness perceptions) did not predict hope and/or will to live and there were no significant interactions between the three (Table 4). The variables were minimally correlated with illness threat. Therefore, a second exploratory regression model was created. Will to live and hope were tested as predictors of heart failure self-care. See Figure 4.

**Table 4:** Outcomes of moderation model 1.

Self-care outcome	Predictor	*β*	SE	*p*	Model *R*^2^
Maintenance	Illness Threat	−0.17	0.10	.055	—
	Hope	0.51	0.10		0.12
	Will to Live	5.18	1.18		0.10
	Interaction (ITxHope/WTL)	—	—	>.01	—
Management	Illness Threat	0.01	0.12	.962	—
	Hope	0.81	0.14		0.14
	Will to Live	9.68	1.50		0.16
	Interaction (ITxHope/WTL)	—	—	>.01	—
Monitoring	Illness Threat	0.22	0.10	.025	—
	Hope	0.65	0.11		0.14
	Will to Live	4.77	1.33		0.06
	Interaction (ITxHope/WTL)	—	—	>.01	—
Self-Efficacy	Illness Threat	−0.21	0.10	.032	—
	Hope	0.74	0.11		0.19
	Will to Live	6.92	1.28		0.14
	Interaction (ITxHope/WTL)	—	—	>.01	—

*β*, standardized regression coefficient; SE, standard error.

Interactions refer to Illness Threat × Hope and Illness Threat × Will to Live,.

all of which were nonsignificant (*p* > .01).

*R*^2^ values come from the **overall model** for each predictor set.

**Figure 4:**
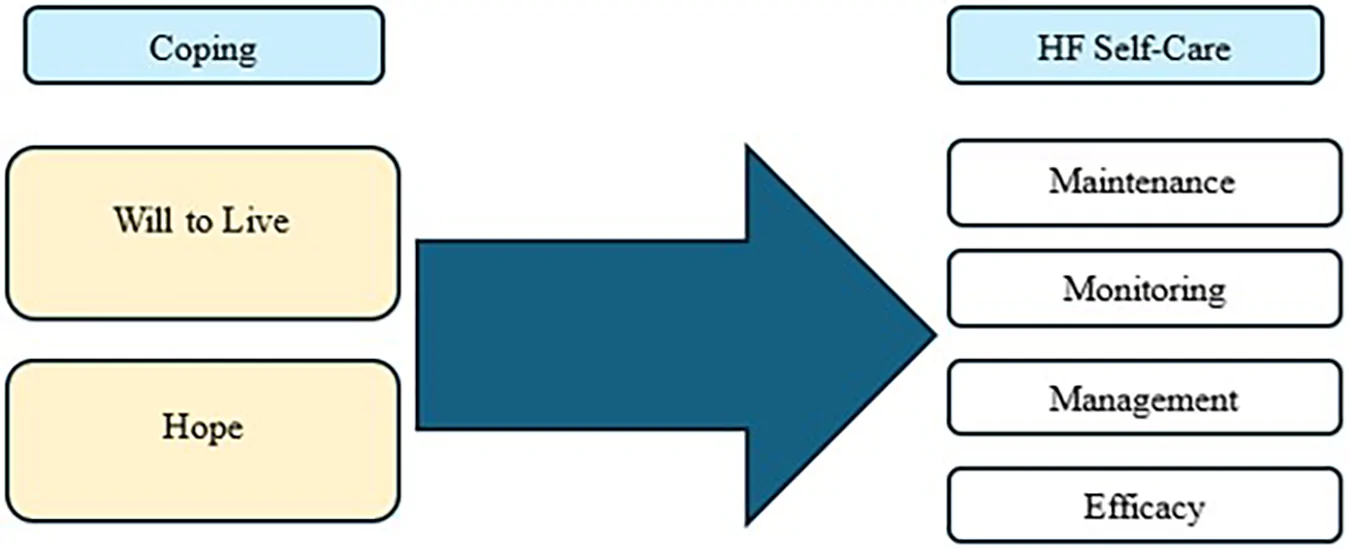
Regression model 2.

### Regression analysis

Regression analysis was conducted based on Model 2. See Table 5. Assumptions were checked and no violations were found.

**Table 5:** Outcomes of regression model 2.

Self-care outcome	Predictor	Hope (*β*,*p*)	Will to Live (*β,p*)	Hope SE	WTL SE	Model *R*^2^
Maintenance	Hope	0.53 (<.001)	–	0.105	–	0.11
	Will to Live	–	5.28 (<.001)	–	1.142	0.09
	Combined	0.42 (<.001)	3.97 (<.001)	0.106	1.151	0.15
Management	Hope	0.81 (<.001)	–	0.136	–	0.14
	Will to Live	–	9.42 (<.001)	–	1.451	0.17
	Combined	0.60 (<.001)	7.55 (<.001)	0.134	1.447	0.24
Monitoring	Hope	0.63 (<.001)	–	0.115	–	0.12
	Will to Live	–	4.07 (.002)	–	1.303	0.04
	Combined	0.57 (<.001)	2.30 (0.08)	0.120	1.292	0.13
Self-efficacy	Hope	0.76 (<.001)	–	0.112	–	0.18
	Will to Live	–	7.31 (<.001)	–	1.238	0.14
	Combined	0.61 (<.001)	5.41 (<.001)	0.111	1.209	0.25

*β*, standardized regression coefficient; SE, standard error.

## Results

### Heart failure self-care (HFSC)

#### Self-care (SC) maintenance

A significant effect of hope was observed on SC maintenance. Higher levels of hope were associated with higher levels of SC maintenance (*p* < .001). When examining the relationship between WTL and SC maintenance, a significant effect was observed. Higher levels of WTL were associated with higher levels of SC maintenance (*p* < .001). Both hope and WTL were included together to determine what unique variance was explained. Both predictors remained significant and the combined model explained slightly more variance than either variable alone (*p* values <0.001, *R*^2^ = 0.15).

#### SC management

A significant effect of hope was observed on SC management. Higher levels of hope were associated with higher levels of SC management (*p* < .001). When examining the relationship between WTL and SC management, a significant effect was observed. Higher levels of WTL were associated with higher levels of SC management (*p* < .001). Both hope and WTL were included together to determine what unique variance was explained. Both predictors remained significant and the combined model explained more variance than either variable alone (*p* values <0.001, *R*^2^ = 0.24).

#### SC monitoring

A significant effect of hope was observed on monitoring. Higher levels of hope were associated with higher levels of SC monitoring (*p* < .001). When examining the relationship between WTL and SC monitoring, a significant effect was observed. Higher levels of WTL were associated with higher levels of SC monitoring (*p* < .001). Both hope and WTL were included together to determine what unique variance was explained. Hope remained significant (*p* < .001) while WTL (*p* = 0.08) failed to reach statistical significance. The combined model explained more variance than either variable alone (*R*^2^ = 0.13).

#### SC efficacy

A significant effect of hope was observed on SC efficacy. Higher levels of hope were associated with higher levels of efficacy (*p* < .001). When examining the relationship between will to live and efficacy, a significant effect was observed. Higher levels of WTL were associated with higher levels of efficacy (*p* < .001). Both hope and WTL were included together to determine what unique variance was explained. Both predictors remained significant and the combined model explained more variance than either variable alone (*p* values <0.001, *R*^2^ = 0.25).

### Bias

Bias was reduced in this study by using valid and reliable measurement tools, establishing that linearity, independence, normality, and equality of variance in the multiple regression models were met.

## Discussion

Across all HFSC domains—maintenance, monitoring, management, and SC efficacy—hope and WTL emerged as consistent and significant correlates. In contrast, illness threat demonstrated weak or non-significant associations with HFSC and did not interact with hope or WTL in predicting HFSC. These findings suggest that coping and dispositional resources may play a more central role in HFSC engagement than illness perceptions alone.

### Illness threat and self-care behaviors

Contrary to expectations derived from the Common-Sense Model of Illness Self-Regulation (CSM), illness threat was not a robust predictor of HFSC in this sample. Although several associations between illness threat and HFSC domains approached statistical significance, none met the prespecified threshold. These findings suggest that perceiving HF as threatening may, on its own, be insufficient to motivate sustained engagement in self-care behaviors. Alhurani and colleagues (33) examined cognitive appraisals in chronic HF and found that harm/loss appraisals—a form of perceived threat—were significantly associated with avoidant emotional coping, rather than task-oriented coping. This directly supports the idea that perceiving HF as threatening can shift coping away from action and toward maladaptive emotional responses unless motivational supports are put in place. A large prospective study by Shimode et al. (34) similarly showed that avoidance-oriented coping under high stress was associated with poorer self-care and higher readmission and mortality rates in HF patients. A scoping review of 35 studies by Alanazi et al. (35) concluded problem-focused coping is less common in patients with higher disease burden or perceived severity while in a more recent review study, Westas and Melnikov (36) reported disease-related fears in advanced HF are associated with restricted activity, anxiety, and reduced motivation, reinforcing the idea that threat perceptions can inhibit action-oriented coping while also pointing to motivation as a key factor.

Within the CSM, illness representations including illness threat are theorized to guide coping behaviors approach and avoidant oriented behaviors; however, the present findings suggest that illness threat may need to be accompanied by sufficient motivational resources to translate awareness into action. Without such resources, individuals may recognize the seriousness of their condition yet remain unable or unwilling to initiate or sustain HFSC behaviors. Conversely, beliefs may also be implicated in threat responses that may lead to failure to engage in treatment-seeking behavior, actions that are erroneous or ineffective in addressing the threat, or denial or minimization of the threat, which may have serious implications for prevention and prognosis of serious illness.

### Hope and will to live as motivational resources for self-care

Hope demonstrated a strong, consistent association across all domains of HFSC. Higher levels of hope were associated with greater HFSC maintenance, monitoring, management, and self-care efficacy, explaining meaningful proportions of variance across models. These findings support Snyder's Hope Theory, which posits that individuals with greater agency and pathways thinking are better equipped to pursue goal-directed behaviors—such as daily medication use, symptom monitoring, and responsive HFSC actions.

Within the CSM framework, hope may function as a key emotional and motivational resource that facilitates adaptive coping responses or serves as an approach-oriented coping response that drives active, action-focused problem-solving rather than avoidant or emotion-focused coping. Hope enables individuals to manage the negative emotions and impaired physical functioning caused by HF allowing them to envision a more positive future while developing perceived pathways to achieve it, thereby fostering resilience in the context of chronic illness or severe distress.

Individuals with higher hope may be better able to interpret HF symptoms as manageable, identify actionable strategies, and persist in self-care despite symptom burden. Importantly, hope's association with self-care efficacy suggests that it may also enhance confidence in one's ability to manage HF, reinforcing self-regulatory cycles over time.

The WTL also demonstrated strong, positive associations across all HFSC domains, and included notable effect sizes with HFSC management and efficacy. These findings extend prior qualitative and conceptual work by providing empirical evidence that WTL is a meaningful correlate of HFSC behaviors. Shrira and colleagues (21) found, in a longitudinal study, that reciprocal relationships exist among WTL and objective and subjective successful aging, which typically require behavioral change to care for and prevent the progression of chronic conditions. Higher WTL at time one predicted higher objective and subjective successful aging at time two (i.e., fewer medical conditions, less disability, less pain symptoms, and higher ratings of subjective successful aging). Higher objective and subjective successful aging at time one predicted higher WTL at time two. In the context of a life-limiting illness such as HF, WTL may serve as a foundational motivational drive that underpins readiness, commitment, and persistence in HFSC activities (21).

Conceptually, WTL may operate at a deeper, more existential level than hope, reflecting an individual's desire to preserve life and find meaning despite illness or make meaning from living with an illness. Within the CSM, WTL may influence both emotional representations and coping orientations, promoting engagement rather than disengagement. The strong associations observed across HFSC domains suggest that individuals with a higher WTL may be more willing to tolerate the burdens of daily self-care to live longer or better.

### Absence of moderation effects

Despite strong associations of hope and WTL, neither construct moderated the relationship between illness threat and HF self-care. This finding suggests that hope and WTL do not necessarily alter how illness threat influences self-care; rather, they appear to exert independent and direct effects on HFSC behaviors. One possible interpretation is that motivational resources such as hope and WTL operate in parallel with illness representations rather than conditionally amplifying or buffering their effects. Alternatively, illness threat may be less salient as a driver of behavior in individuals who have lived with HF long enough to integrate management into their daily lives. In this sample, the mean time in years individuals lived with HF was 5.8 years and the mean illness threat score was 42.10, suggesting this sample was on the border of low to moderate illness threat perception (29). As Riegel and colleagues (25) suggest in the Situation Specific Theory of Heart Failure Self-care, expert decision makers benefit from the experience of the development of intuition, which assists them in making decisions in more dire circumstances such as when there is a time urgency or uncertainty. These individuals are better equipped to recognize patterns which can be useful in symptom interpretation. In such cases, individuals who have had HF and are more expert decision makers, dispositional and motivational factors may carry greater explanatory power than cognitive appraisals of threat. These findings highlight the importance of broadening self-care models to include existential and motivational constructs, particularly in chronic and progressive conditions. Also, power analyses indicated that detecting a small interaction effect (*f*^2^ = .02) with 80% power would require approximately 550 participants. The observed interaction effects in the present study were *f*^2^ = <.005. Thus, the nonsignificant interactions should be interpreted cautiously, as the study was underpowered to detect small moderation effects.

### Implications for theory and practice

The findings of this study have several important clinical implications for the management of HF. First, the consistent and robust associations between hope, WTL, and all domains of heart failure self-care (HFSC) suggest that coping and existential factors are critical targets for clinical assessment and intervention. Traditional HF management has emphasized education, symptom recognition, and adherence strategies; however, these approaches may be insufficient when individuals lack the motivational resources necessary to translate knowledge into sustained self-care behaviors. Routine clinical encounters may benefit from the inclusion of brief, validated assessments of hope and WTL to identify patients at heightened risk of self-care disengagement.

Second, the absence of significant associations between illness threat and HFSC, alongside the strong main effects of hope and WTL, could indicate the need to move beyond fear- or threat-based messaging in patient education. Emphasizing the seriousness of HF without concurrently supporting patients' sense of agency, purpose, or WTL may inadvertently contribute to emotional distress or avoidance rather than action-oriented coping. Clinicians, particularly nurses and advanced practice providers who work closely with patients across the HF illness trajectory, are well-positioned to adopt strengths-based, motivational approaches that foster hope, meaning, and life engagement.

Third, the strong associations observed between WTL and SC management and SC efficacy highlight the relevance of existential and psychosocial dimensions of care in chronic, life-limiting conditions. Integrating palliative care principles earlier in the disease trajectory focused not solely on symptom control, but also on goals of care, meaning, and quality of life may help support patients' WTL and, in turn, their engagement in HFSC behaviors. Interdisciplinary approaches that include mental health professionals, chaplains, and social workers may be particularly beneficial in addressing declines in hope or WTL that can undermine HFSC.

Finally, these findings suggest that personalized HFSC interventions should be tailored not only to symptom burden and cognitive understanding but also to motivational readiness. Interventions designed to enhance hope (e.g., goal-setting, pathways planning, reinforcing agency) and to support WTL (e.g., values clarification, life review, meaning-centered approaches) may improve the effectiveness of self-care support strategies and lead to more sustainable behavior change.

### Future research

Future research is needed to further examine the relationships among illness representations, hope, WTL, and HFSC over time. Longitudinal studies are particularly important for determining causal pathways and examining how changes in hope and WTL influence self-care behaviors as HF progresses. Such designs would also allow for evaluating whether declines in motivational resources precede disengagement from HFSC or emerge as a consequence of symptom burden, disease progression, or repeated hospitalizations. Future studies with a larger sample size could show a stronger association (adequate statistical power) between illness representations, hope, WTL, and HFSC.

Interventional research is also warranted to test strategies specifically designed to enhance hope and WTL in individuals with HF. Randomized controlled trials evaluating hope-based or meaning-centered interventions could help determine whether strengthening these constructs leads to improvements in HFSC maintenance, management, and monitoring, and efficacy. Additionally, future studies should explore whether combining motivational interventions with traditional self-care education yields synergistic effects compared to education alone.

Further theoretical refinement is needed to clarify how hope and WTL operate within the Common-Sense Model of Illness Self-Regulation. Given the absence of moderation effects in this study, future research should examine alternative conceptual roles for these constructs, such as mediators, antecedents, or parallel pathways influencing HFSC. Expanding the CSM to more explicitly incorporate existential and motivational dimensions may enhance its explanatory power for self-regulatory behaviors in chronic, life-limiting illnesses.

Finally, future research should prioritize more diverse and representative samples to assess whether these relationships vary by age, gender, race and ethnicity, socioeconomic status, and HF severity. Qualitative and mixed-methods studies may also provide deeper insights into how individuals conceptualize hope and WTL and how these constructs shape daily decision-making related to HFSC. Such work will be essential for developing culturally responsive, patient-centered interventions that address the full complexity of living with HF.

### Limitations

Limitations of this study include the potential for sampling/selection bias due to the online survey format, which requires participants to have internet access and sufficient literacy. This may not be generalizable and representative of the entire HF population. Additionally, there is a potential for self-report bias due to cognitive limitations and misinterpretations of questions. The recruitment platform (Prolific) may limit the participant variability, as a significant percentage of this study's participants were highly educated, and this may not represent the general HF population. Although the argument could be made that if those who are highly educated (and thus advantaged), like the bulk of the study sample, are having difficulty accessing hope and the will to live as coping mechanisms and then engaging in HFSC others probably are too. Using Prolific as a recruiting tool also limits the ability to substantiate HF as a diagnosis via medical records, physician confirmation, and/or hospital documentation as well as collecting verifiable data on HF functional status, ejection fraction, etc.

Drawing from a more diverse sample could show different results and may make these findings less generalizable to underserved HF populations. A more diverse sample could also improve the reliability scores for the WTL and Hope scales, which in other studies have reached the 0.70 threshold. The study design is limited, representing a specific point in time and potentially influenced by recent events or conditions. Reliability statistics for WTL and Hope in this study were low. The cross sectional design limits the ability to track changes throughout the illness trajectory.

## Conclusion

Contrary to CSM predictions, illness threat perceptions were not associated with HFSC. Traditional HF education often emphasizes illness threat (e.g., hospitalization risk, mortality), yet findings suggest threat alone does not drive HFSC. In contrast, WTL and hope emerged as salient psychosocial resources underpinning HFSC. Assessing and leveraging these motivational factors may enhance the individualization and effectiveness of HFSC interventions. Clinically, HF nurses can incorporate brief assessments of hope and WTL into routine encounters, such as during HF education sessions, transitions of care, periods of symptom escalation, or treatment changes.

## Data Availability

The datasets presented in this article are not readily available, as secondary analysis is underway. Requests to access the datasets should be directed to Stephanie Turrise, turrises@uncw.edu.
